# Evaluation of Fatigue Strength Improvement by CFRP Laminates and Shot Peening onto the Tension Flanges Joining Corrugated Steel Webs

**DOI:** 10.3390/ma8085248

**Published:** 2015-08-19

**Authors:** Zhi-Yu Wang, Qing-Yuan Wang, Yong-Jie Liu

**Affiliations:** 1 Department of Civil Engineering & Mechanics, Sichuan University, Chengdu 610065, China; E-Mails: wangqy@scu.edu.cn (Q.-Y.W.); liuyongjie_2002@163.com (Y.-J.L.); 2 Sichuan Provincial Key Laboratory of Failure Mechanics and Engineering Disaster Prevention & Mitigation, Sichuan University, Chengdu 610065, China

**Keywords:** corrugated plate, fatigue strength, welded joint, carbon fiber, shot peening

## Abstract

Corrugated steel web with inherent high out-of-plane stiffness has a promising application in configuring large span highway bridge girders. Due to the irregularity of the configuration details, the local stress concentration poses a major fatigue problem for the welded flange plates of high strength low alloy structural steels. In this work, the methods of applying CFRP laminate and shot peening onto the surfaces of the tension flanges were employed with the purpose of improving the fatigue strength of such configuration details. The effectiveness of this method in the improvement of fatigue strength has been examined experimentally. Test results show that the shot peening significantly increases hardness and roughness in contrast to these without treatment. Also, it has beneficial effects on the fatigue strength enhancement when compared against the test data of the joints with CFRP strengthening. The stiffness degradation during the loading progress is compared with each treatment. Incorporating the stress acting on the constituent parts of the CFRP laminates, a discussion is made regarding the mechanism of the retrofit and related influencing factors such as corrosion and economic cost. This work could enhance the understanding of the CFRP and shot peening in repairing such welded details and shed light on the reinforcement design of welded joints between corrugated steel webs and flange plates.

## 1. Introduction

Corrugated webs with inherent high out-of-plane stiffness and buckling resistance have been recognized as a substitute to plane webs with stiffeners in welded H-section beams. One of the advantages of corrugated webs is the use of relatively thin web without extra stiffeners which decreases the self-weight and thus reduces construction costs. The corrugated webs are usually welded to the flange plates to form a sort of welded detail. To date, the long-term structural response of corrugated steel web girders of such details has been paid increasing attention [1]. Previous studies have shown that the corrugated steel web girders often failed from fatigue cracks that propagated from the web-to-flange fillet weld toe on the tension flange as the result of geometric irregularity of the corrugated web and its related welded details [2]. As a result, the fatigue behavior of welded joints representing such welded details was highlighted in the recent research work [3]. Also, the methods for the fatigue life improvement of such welded details seem to be of practical significance in the application of corrugated steel web girders in engineering.

Carbon fiber-reinforced polymer (CFRP) composites have been broadly used in various industries to manufacture load-bearing engineering components with high strength/weight ratio. The merits of having good constructability and durability of non-metallic CFRP composites also make them quite promising for the strengthening of steel structures [4]. Fundamental research projects and experimental investigations [5,6,7,8,9,10,11,12] have examined the effectiveness of using CFRP technology in bettering the fatigue behavior of steel girders and welded details of steel bridges. The examination of CFRP in strengthening the welded details with corrugated plate has shown the importance of the reinforcement near the transition curvature between the longitudinal fold and the inclined fold of the corrugated plate [13]. On the other hand, as a highly cost effective surface treatment method, shot peening has been well documented in its ability in the improvement of fatigue strength [14]. This improved behavior is generally ascribed to compressive residual stresses produced in the surface layer of treated parts [15]. The resultant compressive stresses offset the applied or residual tensile stresses and slow the crack growth. Recent research work has demonstrated the effectiveness of shot peening in the improvement of fatigue strength of base materials [16,17] and related structural components [18,19]. Thus, the present paper aims to investigate the use of this surface treatment together with CFRP laminates in bettering the fatigue performance of the welded details joining tension flange and corrugated web.

In this paper, the role of CFRP laminates and shot peening on the fatigue life improvement of such welded details was investigated through a fatigue experimental study. The reinforcement methods are outlined with due consideration of the geometric characteristics of the welded details. The shot peening action on the increase of hardness and roughness of test samples are examined. The effects of both methods on the extension of fatigue life are compared in terms of failure modes, stiffness based damage progress and fatigue life results. Based on the preliminary test results, mathematical regression analysis is reported for the representation of the relations of stiffness and fatigue strength with respect to the variation of the number of cycles. Discussions are also made for the influences of corrosion and economic cost on the retrofit. The results obtained in this study can be taken as a basic reference for the further study involved in the fatigue life extension of related structural details.

## 2. Surface Treatment Method and Experimental Test

Previous studies [3,13] have indicated that the fatigue cracking of welded details are mostly located on the flange plate in the vicinity of the transition curvature between the longitudinal fold and the inclined fold of the corrugated plate. Accordingly, two strengthening methods on the steel surface were employed in this experimental work as:

(1) CFRP strengthening on the full width range. Single-layered CFRP sheets were bonded to both sides of the weld bead close to the transition curvature as well as the bottom surface of the main plate of full width in order to reduce the stress concentration at crack tips. The application of CFRP sheets was started by grinding the surface of the main plate to remove rust until the metal surface was exposed over the area for strengthening. The steel surface was abraded when the surface turns into a dark color using coarse sand paper first (#50~#100), then finish the surface with fine sandpaper (#150). Afterwards, all surfaces were cleaned with cotton cloth soaked in acetone with 5–10 min interval for the evaporation of acetone. A tiny divider was adopted to encircle the area to be bonded to the identical intended thickness. The adhesive was spread on the carbon fiber surface except for the unbounded region using a special spatula tool and checking the adhesive thickness is no less than the diameter of fishing line. The thin layer of adhesive of 0.25 mm thick was also applied on the main plate over the area to be bonded. Subsequently, the carbon fiber sheets were exerted on the steel surface by gently applying pressure so that the excess of the adhesive was squeezed from the edges. The surface for CFRP bonding was finished by taping the carbon fiber sheets so that they are not moved by accident after 24 h curing time. The steel used for all specimens conforms to Q345 steel of the Chinese national standard GB/T 1591-2008. The design weld leg length is 3 mm for all fillet welds. High performance carbon fiber sheet UT70-30 with the thickness of 0.167 mm produced by Toray Industries, Inc, and adhesive Araldite XH 180 manufactured by Huntsman Advanced Materials Co., Ltd. were chosen following the standards of GB 50367-2006 and ISO 2555:1989 respectively. The chemical and mechanical properties of test materials are referred from previous research work [13] and listed in Table 1.

**Table 1:** Chemical composition and mechanical properties of steel, carbon fiber and adhesive.

Type	Chemical Composition (%)					Mechanical Properties			
	C	S_i_	M_n_	P	S	σ_y_ (MPa)	*E*_s_ (MPa)	σ_u_ (Mpa)	δ (%)
Steel (Q345)	0.15	0.22	1.24	0.016	0.010	380	2.1 × 10^5^	510	25
Carbon fiber (UT70-30)	-	-	-	-	-	-	2.52 × 10^5^	4216	1.76
Adhesive (XH 180A/B)	-	-	-	-	-	-	3.5 × 10^3^	55	1.6

(2) Shot peening at the region of transition curvature plus CFRP strengthening on the full width range. Before shot peening, the surface condition was determined by roughness and micro-hardness measurement using profiled geometry. The mechanical grinding and polishing with vibratory polishers were used for initial surface preparation on the steel surface. In practice, this treatment was also combined with that for CFRP bonding. During shot peening process, the steel surface is bombarded with small spherical media named shot. As a result, the small dimples on the steel surface are formed with the local yield in tension. Below the surface, on the other hand, the compressed grains tend to restore the surface to its original shape producing a hemisphere highly stressed location in compression. Thus, a uniform layer of residual compressive stress is developed through overlapping dimples. The shot peening in this test was carried out by means of an injector type system using spherical zirconia-based ceramic shot with project amount 40 kg/min and shot velocity of 50 m/s. All peening work was performed to the coverage of the transition curvature between the longitudinal fold and the inclined fold of the corrugated plate where the fatigue cracks are susceptible to being initiated.

**Figure 1:**
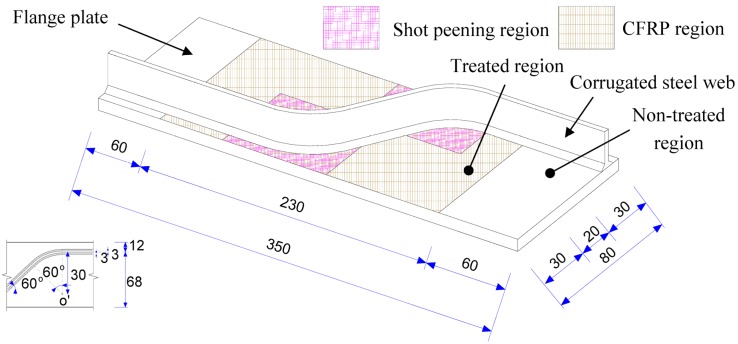
Geometry of a typical test specimen.

**Figure 2:**
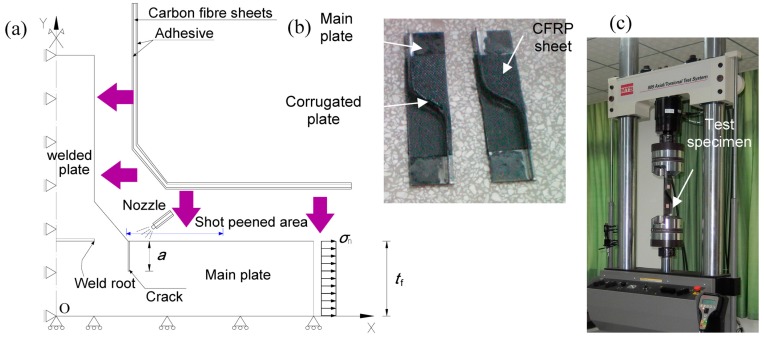
Test specimen with carbon fiber-reinforced polymer (CFRP) + shot peened area. (**a**) Conceptual model for analysis; (**b**) Test specimen; (**c**) Test set-up.

The basic geometry of the test specimen is shown in Figure 1. It was fabricated with corrugated steel plate, containing the longitudinal fold and the inclined fold and the transition curvature, welded to the one side of the flange plate. The CO_2_ shielded semiautomatic Gas Metal Arc Welding (GMAW) was performed for all fillet welds with design weld leg length of 3 mm. This configuration was adopted to represent the welded details of corrugated steel web beams. Since the corrugated steel web inherently possesses sufficient longitudinal flexibility, the longitudinal main (flange) plate was assumed to carry the appreciable part of the tensile stress induced from the overall bending moment. Following previous experimental studies [2,3], the designed geometry of the test joint specimen was comparable to the welded details in the typical corrugated steel web girders. The steel plates used for the test specimens are 6 mm and 3 mm thick for the flange plate and the corrugated steel web plate respectively. The corrugation angle of the inclined fold to the stress in longitudinal direction was taken as 60° in this study. The carbon fiber in the longitudinal direction was arranged to cover the full length of the longitudinal fold and the inclined fold and the transition curvature, representative of a half-unit of the corrugation. Meanwhile, the shot peening area refers to the location in the vicinity of the transition curvature. To clarify the configuration of CFRP strengthened welded details, an explosive plot of a typical cross section is shown in Figure 2a. As the fatigue crack was expected to initiate at the weld toe, the shot peening area was chosen at the surface of the main plate in the vicinity of such location while the CFRP was employed overlapping the surfaces of the corrugated plate and the main plate, as shown in Figure 2b.

The fatigue tests were performed through a MTS 809 servo-hydraulic fatigue testing machine as shown in Figure 2c. Nine fatigue tests concerning the surface treatment were conducted with the comparison with the test data of twelve tests without surface treatment. The one end of the flange plate of the welded joint was secured with 45 mm × 55 mm wedge grips in the test machine to apply longitudinal tensile stress. Hydraulic pressure to the grips was supplied by and adjusted at an external hydraulic grip supply. Constant amplitude sinusoidal stress cycles with the frequency of 8 Hz were conducted during the fatigue test. The target stress range levels between 135 and 200 MPa were chosen and the stress ratio was set at 0.1 for all tests for the purpose of comparison with previous test data [13]. The fatigue life was determined as the test specimen was tested to rupture.

## 3. Experimental Test Results

### 3.1. Surface Condition Measurement

**Figure 3:**
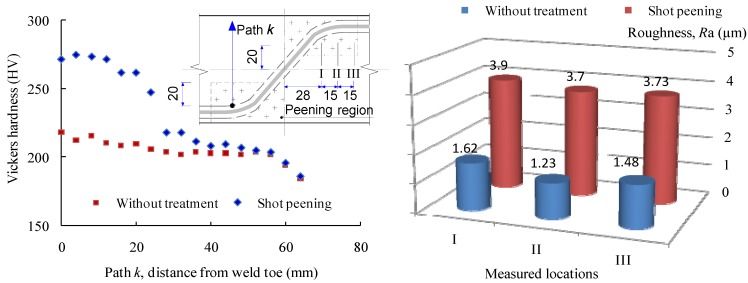
Hardness and roughness comparison of specimens with shot peening and without treatment.

For the purpose of comparing the properties of the surface before and after shot peening, the Vickers hardness and surface roughness (*R*_a_) were measured in the test. The hardness distributions from the weld toe are shown in Figure 3. It can be seen that, in the case of the specimen without treatment, the hardness is slightly greater at the heat affected zone (HAZ) of the weld toe. Away from HAZ, the hardness distribution becomes consistent with approximately 200 HV. In contrast, the use of shot peening significantly increase the hardness with approximately 55 HV enhancement at the treatment location. As the measured location translates outside the peening area, the magnitude of hardness reduces dramatically to that of the specimen without treatment. On the other hand, the surface roughness is significantly increased with the application of shot peening. The average roughness (*R*_a_) for the shot peened specimen is *R*_a_ = 3.78 which is nearly 2.6 times that for the un-treated specimen (*R*_a_ = 1.44).

### 3.2. Failure Observation

**Figure 4:**
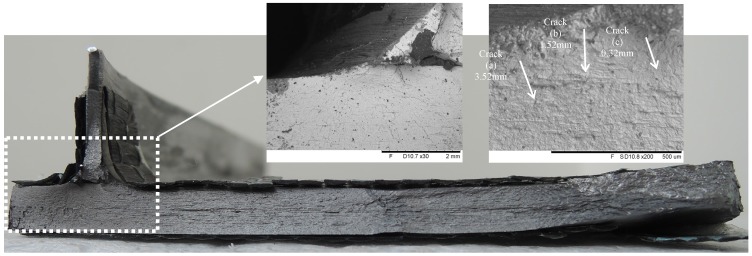
Typical fracture surface at weld toe.

Since the failure mode is similar for all test joints and can be attributed to the stress concentration due to local geometric details, only the typical fracture surface at the location of the transition curvature between the longitudinal fold and the inclined fold of the corrugated plate is plotted in Figure 4 together with crack growth striations under the scanning electron microscope (SEM). Primary crack propagation can be observed originating at the weld toe and propagating into the flange plate. The crack propagation length is gradually reduced when moving away from the weld toe location, *i.e.*, the length of crack (a) near the weld toe is 3.52 mm while these of (b) and (c) is decreased to 1.52 mm and 0.32 mm. The crack propagation pattern on the fracture surface can be idealized as a semi-elliptical shape. Near the edge of the main plate the crack path moved away from the weld toe and tensile fracture occurred locally as evidenced by some discernible necking. By comparing the effect of surface treatment, it is can be observed that the failure modes depend on the adhesive layer failure as well as local separation of some carbon fibers from resin matrix. This indicates that the surface strengthening of CFRP laminates can share some parts of loading carrying capacity prior to the fatigue cracks initiation at the critical location. On the other hand, although the shot peening induced compressive layers are strong so that the crack effect of notches and flaws are shifted to subsurface regions, a similar crack mode was observed in the fatigue tests. Thus, in contrast to the deterioration of the surface, the fatigue crack mode is primarily contributed to by the stress concentration.

### 3.3. Stiffness Degradation Behaviour and Damage Progress

Stiffness is a well defined engineering property, easily measured, and not involved with destruction of the test specimen. During the fatigue loading, damage accumulates with the crack propagation. This is also true for the test welded joints with CFRP strengthening. The damage in the adhesive layer and fiber-resin matrix de-bonding induces degradation of stiffness of CFRP laminates which in turn progressively reduce the stiffness of the welded joints. Regarding this, the stiffness degradation characteristics caused by the fatigue damage are directly related to the damage propagation with the number of cycles. The stiffness at *i*th cycle is given by:

(1) $$R_{i}=\frac{\left({\text{σ}}_{i,\max}-{\text{σ}}_{i,\min}\right)A_{m}}{\delta_{i,\max}-\delta_{i,\min}}$$

where, σ_i,max_ and σ_i,min_ are the maximum and minimum stress components at *i*th cycle; d_i,max_ and d_i,min_ are the deformation components corresponding to σ_i,max_ and σ_i,min_ respectively; *A*_m_ is the plate cross-section area.

**Figure 5:**
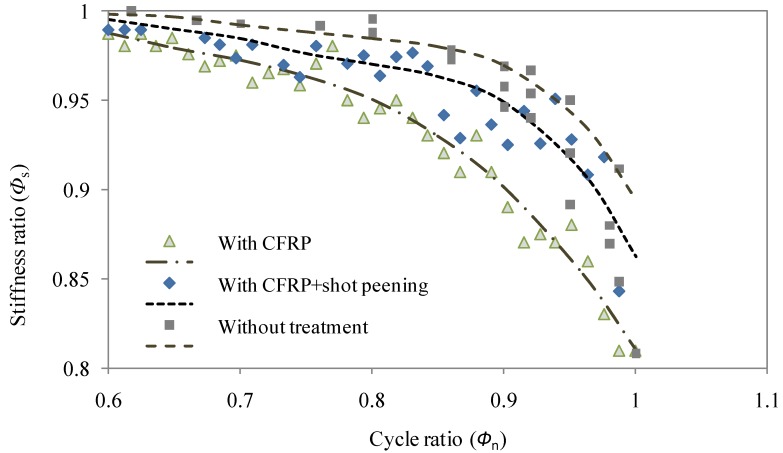
Comparison of relations between stiffness ratio and cycle ratio.

Applying Equation (1) to test data in the representation of the damage progress of the test welded joints, normalized ratios of stiffness (Φ_s_) and the number of cycles (Φ_n_) are defined as:

(2) $$\Phi_{s}=\frac{R_{i}}{R_{0}}$$

(3) $$\Phi_{n}=\frac{N_{i}}{N_{f}}$$

where, *R*_0_ is the stiffness at the initial cycle; *N*_i_ and *N*_f_ are the number of cycles corresponding to *i*th cycle and failure respectively. Given that the significant stiffness reduction took place when Φ_n_ is greater than 0.6, the relation of Φ_s_ with Φ_n_ ranging between 0.6 and 1 is plotted in Figure 5. Also, the damage progress in terms of stiffness can be expressed using the fourth order polynomial regressions for test welded joints as:

(i) For the details without reinforcement and treatment:
  (4) $$\Phi_{s}=-18.6\Phi_{n}^{4}+54.7\Phi_{n}^{3}-60.08\Phi_{n}^{2}+29.13\Phi_{n}-4.256$$
(ii) For the details with CFRP laminate:
  (5) $$\Phi_{s}=2\Phi_{n}^{4}-9.32\Phi_{n}^{3}+13.32\Phi_{n}^{2}-7.84\Phi_{n}+2.65$$
(iii) For the details with surface reinforced with CFRP laminate plus shot peening treatment:
  (6) $$\Phi_{s}=-12.71\Phi_{n}^{4}+33.54\Phi_{n}^{3}-31.91\Phi_{n}^{2}+12.58\Phi_{n}-0.638$$

The resultant correlation coefficients of such fourth order polynomial regressions are 0.96, 0.98 and 0.95 respectively for Equations (4)–(6) respectively. Hence, it is shown the effectiveness in the interpretation of damage progress using such form of expression.

### 3.4. Fatigue Life Results

Recent fatigue design code [20] indicates that for each class of joint, the general equation relating the number of cycles to failure, *N*, and applied stress range, Δ*S*, can be expressed as:

(7) $$N=C{\left(\Delta S\right)}^{-m}$$

where, exponent, *m*, is the slope of the *S*-*N* relation; *C* is the material constant related parameter.

The equation above can also be converted into logarithm form as:

(8) Log(N)=Log(C)−mLog(ΔS)

**Figure 6:**
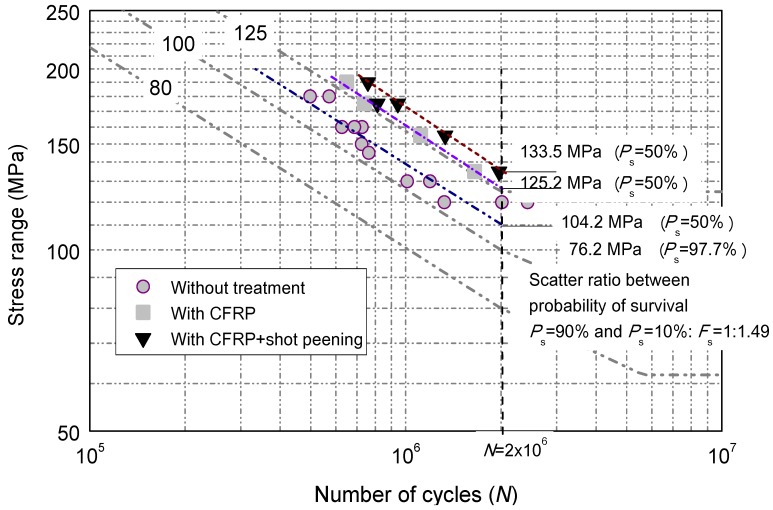
Comparison of fatigue test results.

Following the general fatigue design rule, the test data can be processed based on a statistical analysis to provide the best fit mean *S*-*N* curve by the method of least squares [21]. The fatigue detail categories of JSSC [20] were also added for the purpose of comparison. A graphical presentation in the form of stress range *versus* the number of cycles is shown in Figure 6 with codified detail classes. Referring back to the fatigue test data of the welded joints without reinforcement and treatment [3], the *S*-*N* relation can be given by:

(9) Log(N)=11.576−2.615Log(ΔS)

As a result of regression analysis in this study, the corresponding *S*-*N* relations for test joints can be expressed as:

(i) For the details with surface reinforced with CFRP laminate:
  (10) Log(N)=12.22−2.822Log(ΔS)
(ii) For the details with surface reinforced with CFRP laminate plus shot peening treatment:
  (11) Log(N)=12.51−2.921Log(ΔS)

The comparison of test data with assigned fatigue detail categories by calculating the given constant Log(*C*) of JSSC [20] is also listed in Table 2.

**Table 2:** Comparison of *S*-*N* relations with referred fatigue categories.

Categories/Relations	Standard Deviation	Log(*C*)	CAFL (MPa)
JSSC-*C*	-	12.30	125
JSSC-*D*	-	12.01	100
JSSC-*E*	-	11.74	80
Equation (9)	0.141	11.576	97.68
Equation (10)	0.135	12.22	125.15
Equation (11)	0.139	12.51	133.55

**Figure 7:**
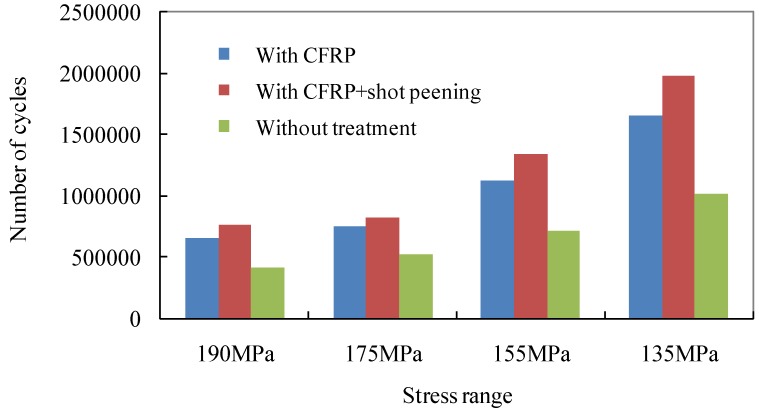
Comparison of fatigue life results of test joints.

To ease the comparison, the fatigue life results corresponding to the stress range at 135 MPa, 155 MPa, 175 MPa and 190 MPa are plotted in Figure 7. It can be seen that the fatigue results of welded joints with CFRP laminates exhibit increase of fatigue life of 45% to 65% while these with the shot peening treatment display a further improvement of roughly 10% to 20%. The latter increase is also equivalent to nearly double the original fatigue endurance of the bare steel joint without reinforcement and treatment.

## 4. Discussion

The study of welded joints presented here is useful for identifying the welded details in corrugated steel web girders of bridge structures. The results of limited fatigue tests provide some novel insights into the contribution of surface treatment on the fatigue life improvement of such details. The distinct fatigue failure mode of the tension flanges joining corrugated steel webs is the crack propagation at the weld toe in the vicinity of the transition curvature of the corrugation. This can be attributed to the presence of transition curvature joining the corrugated plate which inevitably produces abrupt changes of stress flow from the inclined fold and causes local stress concentration. The use of surface treatment methods, *i.e*., CFRP strengthening and shot peening, appeared to retard crack initiation but did not have notable changes of the location of crack propagation caused by local stress concentration (Figure 4). As a result, the typical failure mode of test specimens in this study is similar and comparable to that of the welded details in the corrugated steel web beams as reported in [2,3].

As recognized in the literature that the stiffness of welded details can be significantly increased with CFRP strengthening [12], the stiffness variation during fatigue loading was also concerned in this study. As shown in Figure 5, for the specimens without treatment, the stiffness deteriorates remarkably at the final stage when the cycle ratio is between 0.9 and 1. In contrast, for the specimens with the involvement of CFRP laminates and shot peening, the stiffness deterioration ratio decreases gradually when the cycle ratio is between 0.6 and 1. This indicates that the CFRP laminate may act effectively in the delaying the crack propagation on the tension flanges which in turn produces a moderate loss of stiffness. This is also in agreement with the former reported observation [12] in which the stress decreased with the benefit action of CFRP lamination and thus resulted in the decreased stiffness deterioration. In comparison with the further treatment with shot peening, it can be seen that the welded joint with surface condition of CFRP laminate reinforcement only exhibit less data scatter but lower stiffness (Figure 5). This can be expected as the effect of shot peening in decreasing the deterioration of stiffness with the compensation of the compressive stress introduced by shot peening. However, it is also noted that the effect of such surface treatment is subjected to somewhat fluctuation which can be explained as the results of irregularities of the surface and welded details in the vicinity of the transition curvature where the shot peening was applied. Regarding the correlation expressions, fourth order polynomial regressions in Equations (4)–(6) are shown to attain relatively better correlation than third order polynomial regressions (coefficients of 0.93, 0.92 and 0.90 respectively). Although the goodness of fitting may be further improved using higher order correlation, the fourth order polynomial regressions can be taken as a basic form with less computational cost in the representation of the stiffness deterioration with correlation coefficient no less than 0.95.

The fatigue life data [3] of the welded joints without treatment are reported to be close to the JSSC detail category *D* with constant amplitude fatigue limits (CAFL) of 100. It is noted that, in contrast, the fatigue life is significantly enhanced with the introduction of CFRP laminates on the surface on the tensile plate of the welded joints with visually raising one level of the fatigue detail category, *i.e.*, CAFL from 100 to 125, as shown in Figure 6. The overall fatigue life enhancement is approximately 48% to 65%. This observation can be expected as the extra force carried by the bonded layers on the both sides of the flange plate which produces reinforcement effect, and thus somewhat lessens the tensile action and aids the fatigue life extension. However, such an enhancement is less than that for the reported CFRP strengthened cruciform welded joints [22,23] which resulted in almost 2.14 times improvement of the fatigue life. In this regard, the further enhancement method of shot peening treatment was considered thereafter.

The improving extents of fatigue life induced by shot peening treatment are reported to be almost 30% for base metals [16,17] and 25% for conventional welded joints [18,19]. Similarly, such a treatment also produces roughly 20% to 30% the higher improvements with respect to the CFRP strengthened specimens and results in nearly double the original fatigue endurance of the test specimen without reinforcement and treatment. This can be explained as the introduction of the effect of the compressive stresses induced by shot peening. This effect reduces the tensile stress which attempts to stretch or pull the surface apart and may eventually lead to tensile crack initiation. In other words, the shot peening on the transition curvature of the test welded joint has been confirmed to further delay the initiation of fatigue cracking on the tensile plate and can be adopted along with CFRP laminates for the fatigue life extension of such welded details. In a strict sense, the aforementioned *S*-*N* relations are based on limited test data; however, the constant trend of improvement as a result of adopted methods can be taken as a basis for the further extensive study of fatigue life improvement related to these treatments.

For the sake of considering the engineering application, further investigation of the contribution of environment factors on the structural long-term serviceability is required. This is especially essential for the corrosion which decreases the load-carrying capacity of the members and joints. The corrosion is usually caused by oxidation and galvanic action. Although the CFRP is corrosion resistant minimizing the need for maintenance, the formation of galvanic cell between carbon and steel may still pose a problem of corrosion [24]. The thickness of adhesive and medium properties, such as sizing agent, seawater, *etc.*, may have additional effect on the galvanic corrosion rate [25] thus need further understanding. This requires the novel experimental protocols considering distinct geometric details, *i.e*., the transition curvature, of such welded details.

In the engineering practice, the cost of treatment is another concern. To compare the economic benefit of aforementioned treatment, an economic comparison was developed using an imaginary girder with corrugated steel web. It was assumed that the girder is 24 m long with flange plate area as: 300 mm × 12 mm = 3600 mm^2^. Also, the corrugated steel web is double the geometric size of the details shown in Figure 1 and has the height of 1 m. The results of test specimens with stress range of 155 MPa for this comparison. Based on the test fatigue life data as listed in the second column, the test joint with CFRP laminates and that with CFRP laminate plus shot peening treatment can be converted into *S*_conv_ which are equal to 130 MPa and 122 MPa on the tensile flange respectively. Both types of the girder can be assumed of equal tensile stress to one without treatment on the condition that the flange area can be enlarged by multiplying 155/*S*_conv_. Therefore, the converted flange areas can be computed as 4289 mm^2^ and 4592 mm^2^. By adding the weight of corrugated web, the total weight of the girder, *W*_conv_, can be computed and listed in the fifth column of Table 3. Based on the market steel fabrication price of £ 450/ton, the girder costs can be estimated as £ 1081 and £ 1132 for these with mentioned treatments. This means that, in order to attain aforementioned *S*_conv_ on the tensile flange of the girder, 12% and 17% increase of weight and thus the costs are needed. On the other hand, if only the 1/4 range in the vicinity of the mid-span (supposed to suffer maximum bending moment and tensile stress) are treated, the cost can be evaluated with the supplemented area of contact surface with CFRP and shot peening. Assuming the price of CFRP application as £ 25/m^2^ and that of shot peening cost as £ 32/m^2^, the girder costs can be estimated as £ 1054 and £ 1060 for both cases as listed in Table 3. As in agreement with the findings in [26], therefore, this study also demonstrates that the girders with CFRP laminates and with CFRP laminate plus shot peening treatment can satisfy the requirement of fatigue strength with less fabrication costs and weight saving (12% and 17%).

**Table 3:** Cost comparison of a corrugated steel web girder with or without treatment (Δ*S* = 155 MPa).

Girder Type	*N* (Million Cycles)	*S*_conv_ (MPa)	*A*_ef_ (Mm^2^)	*W*_conv_ (Tonne)	Cost w/o Treatment	Cost after Treatment
w/o treatment	0.71	155 ^†^	3600 ^†^	2.14 ^†^	£ 964 ^†^	£ 964 ^†^
w CFRP	1.12	130	4289	2.40	£ 1081	£ 1054
w CFRP + shot peening	1.33	122	4592	2.52	£ 1132	£ 1060

Notes: Converted stress, *S*_conv_, on the tensile flange is calculated based on Equation (9). *A*_conv_ is the converted flange area which can be calculated as *S*_conv_/155*3600. ^†^ denotes the original value with no treatment.

## 5. Conclusions

The presented study outlined an investigation in the understanding of the use of CFRP laminate and shot peening treatment as the modification of surface conditions in the fatigue life improvement of a specific welded details. The test specimens with shot peening have exhibited much greater hardness and roughness in contrast to these without treatment. The welded details were characterized by fatigue cracking on the flange plate adjacent to the transition curvature between the longitudinal fold and the inclined fold of the corrugated plate. Based on the examination of fracture surface at weld toe, the typical failure mode of test welded joints was discussed. It was shown that the adhesive layer failures as well as local separation of some carbon fibers from resin matrix are the source of failure of test welded joint with CFRP strengthening. The shot peening treatment tends to shift the crack effects to the subsurface regions. The stiffness reduction was employed to characterize the fatigue failure progress. The fourth order polynomial regression has been demonstrated in good representation of the degradation of stiffness with the number of cycles. The shot peening treatment was shown to decrease the tendencies of stiffness deterioration with certain amount of scatter.

The fatigue experimental results have shown that the CFRP strengthening methods adopted in this study greatly enhance the fatigue life of the bare steel joints from CAFL of 100 to that of 125 as codified in JSSC. The method with adding shot peening at the location of transition curvature has further fatigue life improvement of 20% to 30%. This justifies the surface reinforcement with CFRP laminate plus shot peening treatment in the fatigue life enhancement. Finally, the consideration of corrosion in the further work has been discussed and comments have also been addressed for the costs involved in the girders with corrugated steel webs under aforementioned treatment. Notwithstanding this, some further fatigue experimental and analytical studies are still needed to quantify the detailed range of shot peening on such welded details.
